# Pectin Degradation is an Important Determinant for Alfalfa Silage Fermentation through the Rescheduling of the Bacterial Community

**DOI:** 10.3390/microorganisms8040488

**Published:** 2020-03-30

**Authors:** Bing Wang, Zhiqiang Sun, Zhu Yu

**Affiliations:** 1 State Key Laboratory of Animal Nutrition, College of Animal Science and Technology, China Agricultural University, Beijing 100193, China; wangb@cau.edu.cn; 2 College of Grassland Science and Technology, China Agricultural University, Beijing 100193, China; szq141835@sina.com

**Keywords:** 16S rRNA gene, silage, pectinase, bacteria

## Abstract

This study aimed to evaluate the effects of the four kinds of additives on the silage quality and the relevant bacterial community diversity by Illumina HiSeq 16S rRNA sequencing. The four kinds of additives were *Lactobacillus plantarum* (LP), organic acids including gallic acid (GA) and phenyllactic acid (PA), pectin (PEC), and enzymes including pectinase (PEE) and cellulase (CE). After 30 d of fermentation, the pH value was shown to have the lowest value in the PEE and PEC groups, followed by the PA group, and then in CE and GA groups; the highest value of pH was found in both LP and control groups. The ammonia nitrogen concentration was lower in the PEE group compared to the other groups except for the PA group. In the comparisons among the seven groups, *Lactobacillus* was higher in the LP group, *Paracoccus* was higher in the GA group, *Weissella* was higher in the PA group, *Leuconostoc* was higher in the PEC group, *Bacillus*, *Aeromonas*, and *Curvibacter* were higher in the PEE group, and *Coriobacteriaceae_UCG_002* was higher in the CE group compared to the other groups. This study proposed that the addition of PEC and PEE improved the fermentation quality of alfalfa silage compared to other additives by improving the bacterial community of *Leuconostoc*, and *Bacillus* and *Aeromonas*, respectively. Moreover, the enhanced fermentation quality of alfalfa silage by the supplementation of PEC and PEE might be attributed to other unclassified genera. This study provides an implication that pectin degradation is an important determinant for alfalfa silage fermentation through the rescheduling of bacterial community diversity.

## 1. Introduction

Silage is one of the main ingredients and energy and digestible fiber sources in ruminant diets, especially for dairy cattle [1]. Silage making is a bioprocessing ideal method for anaerobic preservation and pretreatment of forage, as it can prolong the stable storage of forage and keep the succulence of grass [1,2]. Alfalfa (*Medicago sativa* L.) is widely used as a forage source in ruminants, but it is difficult to generate high-quality alfalfa silage due to its high buffer capacity and low fermentable carbohydrate [3,4]. Poorly made silage can harbor pathogens, reduce animal performance, cause diseases of cattle, and even potentially endanger human health [5]. It is, therefore, crucial to perform proper quality control by additives’ supplementation including organic acids, lactic acid bacteria (LAB), sugars, and enzymes to generate more lactic acid and lower pH value during ensiling [4].

Alfalfa has a relatively high pectin concentration that ranges from 10.5% to 14.2% (dry matter basis) [6]. It has been reported that the addition of pectinase and cellulase can improve the release of water-soluble carbohydrate (WSC) during the ensiling of alfalfa [7]. The WSC concentration is one of the most important factors during ensiling [4,8]. Thus, we proposed that the release and degradation of pectin from alfalfa itself might be an optional manner to improve the ensiling quality of alfalfa silage due to its abundant pectin. In addition, phenyllactic acid has antifungal activity, which can inhibit the growth of pathogens [7,9]. Gallic acid is well known for its antioxidant activity as a protective antioxidant [10,11]. A recent study found that gallic acid could help to improve the preservation of protein and antioxidant capacity during the ensiling of *Moringa oleifera* leave that has high protein composition [12]. Thus, we hypothesized that the addition of the enzymes and chemical additives may have efficient roles in improving the fermentation quality of alfalfa silage.

Bacterial communities play a crucial role for ensiling forage during fermentation, which produces various metabolites and involves many types of microorganisms [13]. It has been proven that the addition of additives affected the ensiling quality by changing the profiling of silage bacterial communities [14,15]. Therefore, the main purpose of the current study was to evaluate the fermentation quality and the relevant bacterial community diversity when the four kinds of additives (LAB, sugar, organic acids, and enzymes) were introduced for ensiling alfalfa, and to see which additive showed more determining roles.

## 2. Materials and Methods

### 2.1. Experimental Design

The experiment was conducted in June, 2019 at one rural land field (Nanyang, Henan, China). Alfalfa was planted in fields of loam soil, and no herbicides or fertilizers were applied. Alfalfa was harvested at the squaring stage after the first cutting. After cutting and wilting for several hours, the alfalfa was chopped into 1-to-2-cm segments with a forage cutter (Lingong Machinery, Shandong, China). The dry matter of the alfalfa material before ensiling was 27.3%. The chopped forage was sampled and analyzed for chemical composition. The concentrations of crude protein, neutral detergent fiber, acid detergent fiber, and WSC in alfalfa before ensiling were 17.1%, 32.8%, 23.4%, and 4.77% of the dry matter basis, respectively. The seven treatments, including a control (CON), *Lactobacillus plantarum* (LP, 1 × 10^6^ cfu/g of fresh matter), gallic acid (GA, 0.5% of fresh matter), phenyllactic acid (PA, 0.5% of fresh matter), pectin (PEC, 65% purity, from citrus, 2% of the fresh matter), pectinase (PEE, 50 U/g, from *Aspergillus niger*, 0.05% of fresh matter), and cellulase (CE, 10,000 U/g, from *Trichoderma reesei*, 0.05% of fresh matter), were used for silage and evaluated with three replicates each of alfalfa before ensiling. The LAB strain was isolated from alfalfa (Beijing, China) silage and identified as *Lactobacillus plantarum* (GenBank accession number: WCFS1) by 16S rRNA gene sequencing [16]. The strain was inoculated into liquid optimized medium for LAB and cultivated for 20 h. Then, the *L. plantarum* cells were collected by centrifugation at 5000 g for 10 min at 4 °C. The bacterial powder was obtained by freeze drying the cell pellets mixed with non-fat dry milk. Before use, the inoculum was prepared by resuspending the powder in deionized water to a concentration of 1 × 10^12^ cfu/g. The added dose of pectinase and cellulase was according to a previous study of Nadeau et al. [7]. The additive was mixed with 2 mL of sterile distilled water and sprayed onto the 200 g chopped alfalfa (only 2 mL of sterile distilled water for CON). The mixed material (200 g) was packed into polyethylene oxygen isolation plastic film bags (Hiryu KN type, dimensions: 180 mm × 260 mm; thickness: 0.2 mm; Embossed Food saver bag; Asahikasei, Tokyo, Japan). Then, the bags were sealed with a vacuum sealer (BH950; Matsushita, Tokyo, Japan) and stored at ambient temperature (22–25 °C) in dark conditions for 30 d of ensiling.

### 2.2. Sample Collection and Measurements

The silage sample (20 g) was mixed with distilled water (180 mL), homogenized in a blender for 30 s, and filtered through four layers of cheesecloth [17]. The pH was measured immediately using a pH meter (PHS-3C, INESA Scientific Instrument, Shanghai, China). The concentrations of lactic acid, acetic acid, propionic acid, and butyric acid were measured by high-performance liquid chromatography (HPLC, Shimadzu, Tokyo, Japan). The contents of ammonia nitrogen were determined according to Broderick and Kang [18].

### 2.3. DNA Extraction and Sequencing

The microbial pellet was obtained from silage according to the procedure from a previous study [19]. The alfalfa silage is a kind of fermented product of feed, which is similar to the stool sample. Thus, the microbial DNA was extracted using the E.Z.N.A. stool DNA kit (Omega Biotek, Norcross, GA, U.S.) according to the manufacturer’s protocols. The 16S rDNA V3-V4 region of the eukaryotic ribosomal RNA gene was amplified by PCR using primers 341F: CCTACGGGNGGCWGCAG and 806R: GGACTACHVGGGTATCTAAT. Amplicons were extracted from 2% agarose gels and purified using the AxyPrep DNA Gel Extraction Kit (Axygen Biosciences, Union City, CA, USA) according to the manufacturer’s instructions and quantified using QuantiFluor-ST (Promega, Madison, WI, USA). Purified amplicons were pooled in equimolar ratios and paired-end sequenced (2 × 250) on an Illumina platform by Hiseq2500 PE250 (Illumina, San Diego, CA, USA) according to the standard protocols. The raw reads were deposited into the NCBI Sequence Read Archive (SRA; http://www.ncbi.nlm.nih.gov/Traces/sra/) database (Accession Number: SRP250821).

### 2.4. Bioinformatic Analysis

Raw data containing adapters or low-quality reads would affect the following assembly and analysis. The raw sequences were selected according to Wang et al. [20]. Paired-end clean reads were merged as raw tags using FLSAH (v 1.2.11) with a minimum overlap of 10 bp and mismatch error rates of 2%. Noisy sequences of raw tags were filtered by the QIIME (V1.9.1) pipeline under specific filtering conditions to obtain clean tags. To perform a reference-based check for chimera, the clean tags were compared against the reference Gold database (r20110519; http://drive5.com/uchime/uchime_download.html) using UCHIME Algorithm (http://www.drive5.com/usearch/manual/uchime_algo.html).

All chimeric tags were removed to obtain effective tags, then the effective tags were clustered into operational taxonomic units (OTUs) of ≥97% similarity using the UPARSE pipeline. The OTUs were extracted according to the minimum abundance. A Venn analysis was performed to identify unique and common OTUs Between the seven groups. The analysis of the taxonomy assignment of representative sequences was performed by a naive Bayesian model using an RDP classifier that was based on the SILVA (version v123) database. Alpha diversity indices including Sobs, Shannon, Simpson, Ace, Chao, and Coverage were calculated in QIIME. The OTU rarefaction curve and rank abundance curves were plotted in QIIME. Welch’s *t*-test and a Wilcoxon rank test were performed for the alpha index comparisons between two groups. Tukey’s honestly significant difference (HSD) test and a Kruskal-Wallis H test were performed to compare the alpha indices among groups. For comparisons of the seven groups, the permutational multivariate analysis of variance (PERMANOVA) based on bray-curtis distances were performed using the vegan package in the R programming environment [21,22]. Multivariate statistical analyses, including a principal coordinates analysis (PCoA) of bray-curtis distances, were calculated. The functional genes of the bacterial communities were predicted using Tax4Fun. The non-strict version of LEfSe (Linear discriminant analysis Effect Size) was utilized to select and demonstrate the differentially abundant taxonomies among the groups by coupling a nonparametric factorial Kruskal-Wallis sum-rank test for statistical significance with additional tests assessing biological consistency and the relevance of effects. Bacteria with linear discriminant analysis (LDA) scores greater than 3 were speculated to have a different abundance.

### 2.5. Correlations Analysis

To explore the functional correlation between the rumen bacterial changes and fermentation quality of alfalfa silage, a Spearman’s rank correlation matrix was generated by calculating the Spearman’s correlation coefficient among the taxa that were affected by the diet type (at the genus level, *p* < 0.05) and fermentation characteristics in the R program, and only connections with a *p*-value of less than 0.01 and r > 0.55 or r < −0.55 were retained. These correlations were visualized using the R program pheatmap package. To detect the relationship of fermentation organic acids with samples, redundancy analysis (RDA) was performed at the genus level using the R program (https://www.r-project.org) vegan package, with organic acid proportions considered as explanatory variables.

### 2.6. Statistical Analysis

The fermentation characteristics were analyzed using one-way Analysis of Variance (ANOVA) analysis and Duncan’s multiple range tests based on the GLM procedure of SAS (version 9.2, SAS Institute Inc., Cary, NC, USA). Significance was declared at *p* < 0.01.

## 3. Results

### 3.1. Fermentation Characteristics of Silage

The PEE and PEC groups had the lowest pH value, followed by the PA group, and then by the CE and GA groups, and the highest pH was found in the LP and CON groups (Table 1). The ammonia nitrogen concentration was lower in the PEE group compared to the other groups except for the PA group. The ammonia nitrogen concentration of CON was in the highest value compared to other groups, followed by LP, CE, and GA. The PEC group had the highest lactic acid concentration and the CON had the lowest value. The acetate proportion was greater in the LP and GA groups compared to the PA, PEC, and PEE groups. The propionate concentration was higher in GA compared to other groups except for PA.

### 3.2. Bacterial Profiles in the Silage

**Figure 1:**
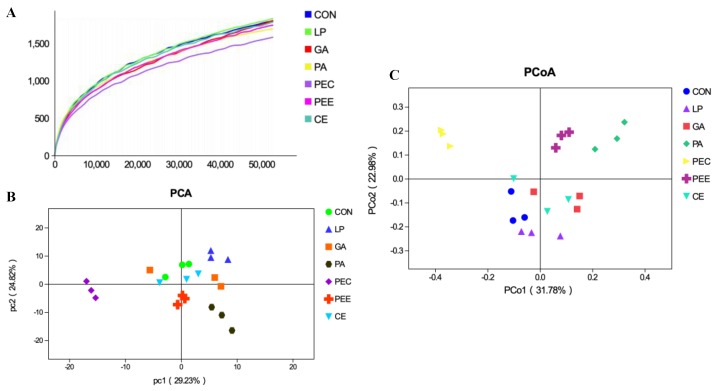
The rarefaction curves of the 16S rRNA gene reads derived from the Sobs index at the operational taxonomic unit (OTU) level after normalization (**A**), principal component analysis (PCA) plot (**B**), and principal coordinate analysis (PCoA) plot (**C**), showing variation in bacterial community structure among the seven alfalfa silage treatments with no additive (CON), *Lactobacillus plantarum* (LP), gallic acid (GA), phenyllactic acid (PA), pectin (PEC), pectinase (PEE), and cellulase (CE). Each point represents an individual sample.

**Figure 2:**
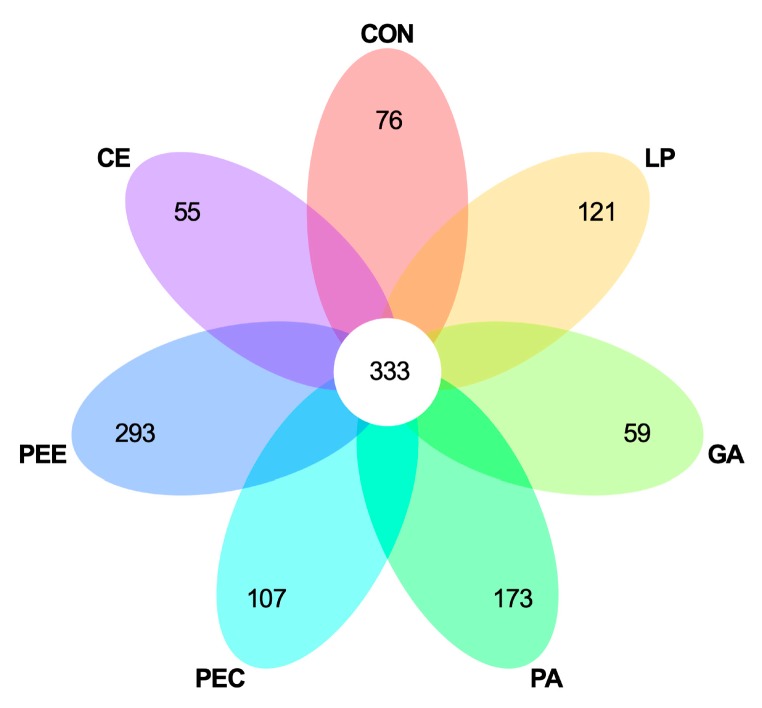
Venn diagram illustrating overlap of microbial operational taxonomic units (OTUs) at 3% dissimilarity level across the treatments.

In total, 2,025,710 raw reads were obtained for the bacterial 16S rRNA genes in the seven groups. After screening, 1,866,479 effective tags were obtained, accounting for 92.15% of the raw reads. The rarefaction curves showed clear asymptotes, and the number of reads was the same for all samples after normalization (Figure 1A). The Good’s coverage value for all samples was greater than 99.1%. The results of the PCA and PCoA with bray distances indicated that the treatment groups of PA, PEC, and PEE were largely separated from other groups including CON, LP, GA, and CE at the OTU level (Figure 1B,C). There were 1217 OTUs that were identified in the silage of the seven groups, among which 333 OTUs were found in all the seven groups and accounted for 27.4% of the total OTUs (Figure 2). There were 76, 121, 59, 173, 107, 293, and 55 OTUs that were identified individually in CON, LP, GA, PA, PEC, PEE, and CE, respectively (Figure 2). For the alpha diversity analysis, a similar level of species richness existed among the seven groups based on the Sobs, Simpson, Ace, and Chao index analyses, which indicated that there was a similar tendency of diversity and uniformity among the seven groups (Table 2). The Shannon index analysis indicated that there were diversity and uniformity between CON and GA, between CON and PEC, and between PEC and PEE.

### 3.3. Silage Bacteria Changes

**Figure 3:**
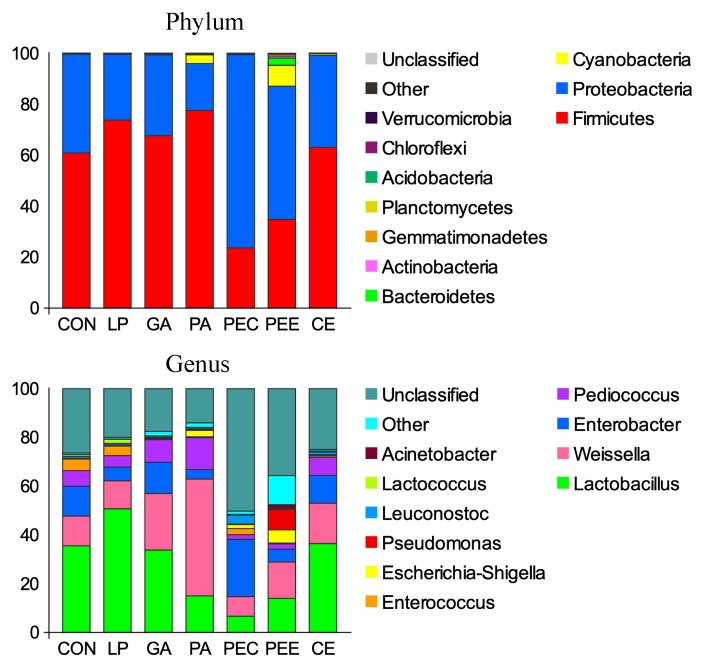
The relative abundance of bacteria community proportions at the phylum and genus level across the treatments (as a percentage of the total sequence).

In total, 27 bacterial phyla were identified in the silage samples. Among these phyla, *Firmicutes*, *Proteobacteria*, and *Cyanobacteria* had relatively high abundances, with mean abundance levels of 57.3%, 39.9%, and 1.92%, respectively (Figure 3). There were 327 bacterial taxa identified at the genus level, and 16 genera were present in all samples, which was indicative of the core microbiome in this study (Figure 3). *Lactobacillus* (27.4%), *Weissella* (19.2%), *Enterobacter* (10.7%), *Pediococcus* (6.43%), *Enterococcus* (1.85%), *Escherichia-Shigella* (1.54%), *Pseudomonas* (1.32%), *Leuconostoc* (1.03%), *Lactococcus* (0.56%), and *Acinetobacter* (0.25%) were considered the top 10 high-abundance taxa (Figure 3).

**Figure 4:**
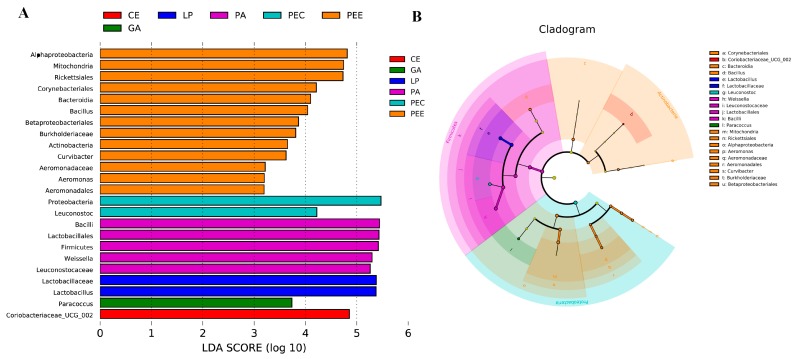
The core specific bacterial biomarker. LEfSe (Linear discriminant analysis Effect Size) analysis shows differentially abundant bacteria communities across the six additives groups as biomarkers determined using the Kruskal-Wallis test (*p* < 0.05) with a linear discriminant analysis (LDA) score >3.0 (**A**). This cladogram is color-coded; in brief, the orange bars, for example, represent the bacteria with the highest abundance in pectinase (PEE) compared with those in the other groups. It shows a representation of the differentially abundant bacteria (highlighted by small circles and by shading) among the six additives groups (**B**). There are six layers from the inside of this plot to the outside, corresponding to six levels of the taxonomy (kingdom, phylum, class, order, family, and genus). The size of each node represents their relative abundance.

The non-strict version of LEfSe was used to determine the bacteria most likely to explain the differences among the different additives’ treatments (Figure 4). *Lactobacillus* was higher in the LP group, *Paracoccus* was higher in the GA group, *Weissella* was higher in the PA group, *Leuconostoc* was higher in the PEC group, *Bacillus*, *Aeromonas*, and *Curvibacter* were higher in the PEE group, and *Coriobacteriaceae_UCG_002* was higher in the CE group compared to the other groups.

### 3.4. Correlation Analysis

**Figure 5:**
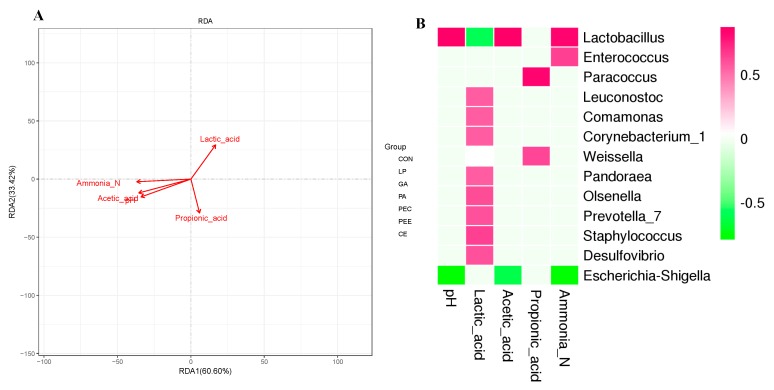
Relationships of fermentation characteristics with silage bacterial community. Redundancy analysis (RDA) of bacterial data (symbols) and fermentation characteristics (arrows) (**A**). Correlation matrix between the fermentation characteristics and the enriched bacteria at the genus level (**B**). Positive correlations are shown in red, and negative correlations are shown in green. Color intensity is proportional to the correlation values (*r* ≥ 0.59) within the seven groups.

RDA revealed that the bacterial community structure was affected by fermentation characteristics (including pH, lactic acid, acetic acid, propionic acid, and ammonia nitrogen), in which the length and angle of each arrow represents its degree and direction of correlation to the bacterial community in the RDA (no permutations), respectively (Figure 5A). As shown in Figure 5A, pH (*p* = 0.001), lactic acid (*p* = 0.004), acetic acid (*p* = 0.001), propionic acid (*p* = 0.016), and ammonia nitrogen (*p* = 0.001) significantly affected the bacterial community structure. The samples that belong to the PEE and PEC groups, located in the positive direction of the lactic acid arrow, were positively related to lactic acid, whereas most of the other samples were negatively related to lactic acid.

It was found that the fermentation indices were related to the silage bacteria community (Figure 5B). In detail, *Lactobacillus* (*r* = 0.86, *p* < 0.01) was positively correlated with the pH value and *Escherichia-Shigella* (*r* = −0.79, *p* < 0.01) was negatively correlated with the pH value. *Lactobacillus* (*r* = −0.58, *p* < 0.01) was negatively correlated with lactic acid, and *Leuconostoc* (*r* = 0.56, *p* < 0.01), *Comamonas* (*r* = 0.57, *p* < 0.01), *Corynebacterium_1* (*r* = 0.56, *p* < 0.01), *Pandoraea* (*r* = 0.56, *p* < 0.01), *Olsenella* (*r* = 0.62, *p* < 0.01), *Prevotella_7* (*r* = 0.60, *p* < 0.01), *Staphylococcus* (*r* = 0.65, *p* < 0.01), and *Desulfovibrio* (*r* = 0.60, *p* < 0.01) were positively correlated with lactic acid. *Lactobacillus* (*r* = 0.87, *p* < 0.01) was positively correlated with acetate and *Escherichia-Shigella* (*r* = −0.61, *p* < 0.01) was negatively correlated with acetate. *Paracoccus* (*r* = 0.83, *p* < 0.01) and *Weissella* (*r* = 0.62, *p* < 0.01) were positively correlated with propionate. *Lactobacillus* (*r* = 0.82, *p* < 0.01) and *Enterococcus* (*r* = 0.64, *p* < 0.01) were positively correlated with ammonia nitrogen, and *Escherichia-Shigella* (*r* = −0.76, *p* < 0.01) was negatively correlated with ammonia nitrogen.

## 4. Discussion

Silage bacterial inoculants, chemical additives, and mixed additives are known for their positive effects such as improving fermentation, increasing nutrients recovery, extending aerobic stability, and mitigating the pathogenicity of silage [4]. For the uneasily fermented materials, the most important manner is to improve their fermentation quality. It has been reported that the pectinase or cellulase appears to contribute to the fermentation of alfalfa silage [7]. In our study, the addition of pectin or pectinase modified the fermentation characteristics including pH and lactic acid concentration. However, the bacterial function underlying the contribution of additives to ensiling alfalfa still needs to be clarified. From the subsequent bacterial sequence data, we found the diversity of the bacterial community contributed to the efficient roles of these additives.

The dominant strains in the alfalfa silage belong to the genera *Lactobacillus*, *Enterococcus*, *Weissella*, and *Streptococcus* in previous studies of alfalfa silage [23,24], which was a little different from the present study. The dominant three genera bacteria across the seven groups were *Lactobacillus*, *Weissella*, and *Enterobater*. The LP belongs to the genus *Lactobacillus*, and the significantly greater level of *Lactobacillus* in the LP group confirmed the supplementation and colonization of LP in alfalfa silage. However, in the current study, the *Lactobacillus* was positively correlated with the pH value and ammonia nitrogen, which was contrary to the general common findings. We estimated that the contribution of LP in decreasing pH and ammonia nitrogen might be less significant than other silage bacteria. From the view of genera classification, more unclassified bacteria were found in the PEC and PEE groups (Figure 3), which might contribute to the improved fermentation qualities. Thus, we proposed that the contribution of decreasing pH value of alfalfa silage might be due to other key microorganisms, except for *Lactobacillus* during ensiling.

The genus *Weissella*, which belongs to LAB, is assigned to the phylum *Firmicutes* [25], which was greater in the PA group compared to other groups. It was reported that the strains of *Weissella cibaria* FMF4B16 belong to the genera *Weissella* and have strong inhibitory activity against food molds [26]. Then, the antifungal compounds such as phenyllactic acid, 2-hydroxy-4-methylpentanoic acid, and other organic acids were investigated to be responsible for the antifungal activity of *Weissella*. Thus, there existed linkage between phenyllactic acid and *Weissella*, and we proposed that the phenyllactic acid might contribute to the growth of *Weissella*, which need more research to clarify it. In addition, it was found that the dominant genera *Weissella* was found in the more easily fermented forages such as corn, sorghum, and forage paddy rice silages [27]. Thus, the dominant and increased abundance of genera *Weissella* in the PA group might indicate the well-fermented alfalfa silage.

The genus *Paracoccus* is facultative aerobic, Gram-stain-negative, short rod-shaped, catalase-positive, and oxidase-positive bacteria belonging to the class *Alphaproteobacteria*. Members of the genus *Paracoccus* can exist in various environments such as air [28], freshwater [29], and agricultural field [30]. It was reported that one species of *Paracoccus*, *Paracoccus jeotgali la sp.* nov., can utilize pectin, acetoacetic acid, myoinositol, propionic acid, glycyl-L-proline, α-keto-glutaric acid, citric acid, L-malic acid, and butyric acid [31]. In addition, it was reported that gallic acid has hydrogen-scavenging activity [32]. Borges et al. [33] reported that gallic acid induced irreversible changes in *E. coli*, *P. aeruginosa*, *S. aureus*, and *L. monocytogenes* membrane properties by the interaction of gallic acid with bacterial cytoplasmic membrane causing hydrophobicity changes, a decrease in negative surface charge, and local rupture and pore formation with leakage of intracellular constituents. Thus, the results of the current study may offer a new finding: that the addition of gallic acid has the ability to induce the accumulation of *Paracoccus* by utilizing gallic acid.

*Leuconostoc*, positively correlated with lactic acid, was the dominant genera in the PEC group alfalfa silage [27]. It was found that *Leuconostoc* was the major bacterial genus present from the initial to the middle stages of fermentation [34]. All the bacteria belonging to *Leuconostoc* could utilize D-fructose [35]. *Leuconostoc* was enhanced by the supplementation of pectin in the current study, which might indicate that the pectin is an important substrate for the growth of *Leuconostoc* during alfalfa ensiling.

*Bacillus* species can produce compounds displaying antifungal activity [36]. *Bacillus amyloliquefaciens* has been reported to be a promising candidate for new pharmaceutical agents and probiotics [37]. *Bacillus* has been proven to produce bacteriocins, bacteriocin-like substances, and lipopeptides to exert antifungal effects [38]. *Bacillus subtilis* SPB1 lipopeptides exhibit surfactant properties and antifungal activity [39]. *Bacillus velezensis* was evaluated as the antagonistic properties towards toxigenic molds in silage conditions [40], indicating the bioactive roles of PEE in promoting the growth of *Bacillus* to exert the antifungal properties. *Aeromonas* was evaluated for pseudomonads fermenting carbohydrates with the production of carbon dioxide and hydrogen [41]. It was reported that *Aeromonas caviae* is potentially vital for acidogenesis in anaerobic digestion, and can be grown on glucose [42]. Thus, the increased abundance of *Aeromonas* in the PEE group might be due to the release of carbohydrates such as pectin or other easily fermented sugars by the pectinase. However, we did not explain the linkage between *Curvibacter* and pectinase as well as the contribution of *Curvibacter* to the fermentation of alfalfa silage.

The results from the present study support the hypothesis of adding specific additives can improve alfalfa silage fermentation by manipulating the bacterial community. However, we believe that the limitation of this study was mainly due to the relatively low number of samples. The function of the differential genera bacteria should be studied for their targeted efficient strains, such as the growth of *Bacillus* and *Aeromonas* in silage. Moreover, we still found many unclassified genera bacteria in the PEC and PEE groups. Given the existence of methods to manipulate the inoculants, the sequencing data should be used for further screening and enrichment culture [43]. Manipulating the levels of species or even strains belonging to the *Weissella*, *Leuconostoc*, *Bacillus*, and *Aeromonas*, as well as other unclassified bacteria that potentially have a close connection to the anaerobic fermentation of alfalfa silage, which may help to improve the quality of alfalfa silage and the development of associated feed industry.

## 5. Conclusions

Overall, the PEC and PEE groups had more efficient roles in contributing to the fermentation of alfalfa silage by improving lactic acid concentration and decreasing pH value and ammonia nitrogen proportion compared to the CE, GA, PA, and LP groups. It was proposed that the addition of PEC and PEE improved the fermentation quality of alfalfa silage by improving the growth of *Leuconostoc*, and *Bacillus* and *Aeromonas*, respectively. The differential bacterial community and unclassified bacteria provided new information on screening targeted or unknown functional bacterial inoculants for modulating alfalfa silage quality.

## Figures and Tables

**Table 1:** Fermentation characteristics of alfalfa silage prepared with different additives.

Items ^2^	Treatments ^1^							SEM ^3^	*p*-Value
	CON	LP	GA	PA	PEC	PEE	CE		
pH	5.17 ^a^	5.09 ^a^	4.86 ^b^	4.47 ^c^	4.29 ^d^	4.15 ^d^	4.85 ^b^	0.033	<0.001
AN, % TN	23.3 ^a^	21.2 ^ab^	16.0 ^c^	9.18 ^ed^	11.7 ^d^	8.40 ^e^	19.4 ^b^	0.59	<0.001
Lactic acid, g/kg DM	75.9 ^c^	77.6 ^bc^	78.0 ^bc^	81.2 ^abc^	112.1 ^a^	107.9 ^ab^	96.5 ^abc^	6.42	<0.001
Acetate, g/kg DM	32.4 ^abc^	48.6 ^a^	43.1 ^a^	15.2 ^cd^	11.4 ^d^	16.6 ^bcd^	35.8 ^ab^	3.89	<0.001
Propionate, g/kg DM	20.3 ^c^	22.3 ^bc^	34.9 ^a^	30.1 ^ab^	19.1 ^c^	16.2 ^c^	17.9 ^c^	1.87	<0.001

^a–e^ Means within a row with different superscripts differ (*p* < 0.01). ^1^ CON = control, LP = *Lactobacillus plantarum*, GA = gallic acid, PA = phenyllactic acid, PEC = pectin, PEE = pectinase, CE = cellulase. ^2^ DM = dry matter, AN = ammonia nitrogen, TN = total nitrogen. ^3^ SEM = standard error of mean.

**Table 2:** Diversity statistics of the bacterial community during ensiling.

Item ^1^	Sobs	Shannon	Simpson	Chao	Ace
CON	1095	5.96	0.96	1808	1842
LP	1102	5.74	0.94	1844	1863
GA	1022	5.49	0.94	1813	1746
PA	1073	5.67	0.95	1702	1710
PEC	933	5.48	0.94	1591	1612
PEE	1016	6.23	0.96	1749	1765
CE	1066	5.74	0.96	1792	1828
SEM ^2^	18.7	0.067	0.003	32.8	29.1
*p*-value	0.25	0.03	0.26	0.33	0.24

^1^ CON = control, LP = *Lactobacillus plantarum*, GA = gallic acid, PA = phenyllactic acid, PEC = pectin, PEE = pectinase, CE = cellulase. ^2^ SEM = standard error of mean.
