# Correction: Wang, B., et al. Pectin Degradation Is an Important Determinant for Alfalfa Silage Fermentation through the Rescheduling of the Bacterial Community. *Microorganisms* 2020, *8*, 488

**DOI:** 10.3390/microorganisms8050769

**Published:** 2020-05-20

**Authors:** Bing Wang, Zhiqiang Sun, Zhu Yu

**Affiliations:** 1 State Key Laboratory of Animal Nutrition, College of Animal Science and Technology, China Agricultural University, Beijing 100193, China; wangb@cau.edu.cn; 2 College of Grassland Science and Technology, China Agricultural University, Beijing 100193, China; szq141835@sina.com

The authors wish to make the following correction to this paper [1]. The dots of the samples in the Figure 5 are missing, and as such, the authors would like to replace the original Figure 5: 
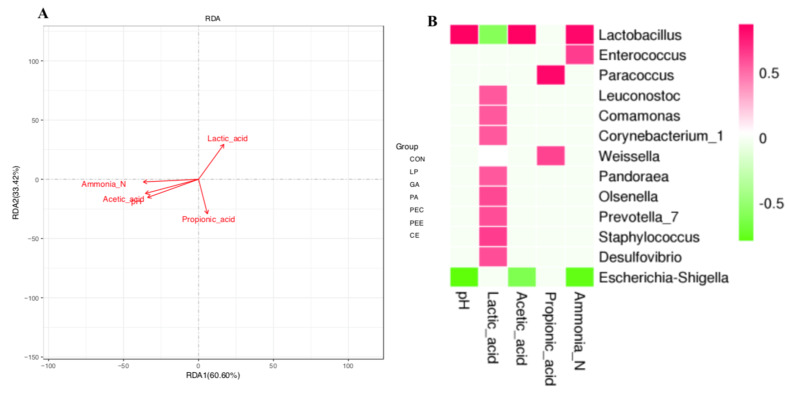
 with a new, updated Figure 5:

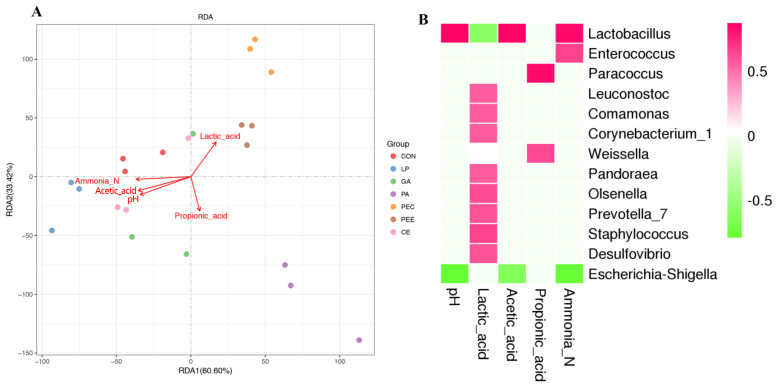



The manuscript will be updated, and the original will remain online on the article webpage, with a reference to this correction. The authors would like to apologize for any inconvenience caused to the readers by these changes.
